# Bona fide receptor for hepatitis B and D viral infections: Mechanism, research models and molecular drug targets

**DOI:** 10.1038/s41426-018-0137-7

**Published:** 2018-07-26

**Authors:** Yueran Yu, Shangda Li, Weifeng Liang

**Affiliations:** 1 State Key Laboratory for Diagnosis and Treatment of Infectious Diseases, Collaborative Innovation Center for Diagnosis and Treatment of Infectious Diseases, The First Affiliated Hospital, College of Medicine, Zhejiang University, Hangzhou, 310003 China; 2 Shengzhou People’s Hospital, Shengzhou Branch of the First Affiliated Hospital of Zhejiang University, Shengzhou, 312400 China; 3 Department of Psychiatry, The First Affiliated Hospital, College of Medicine, Zhejiang University, Hangzhou, 310003 China

## Abstract

Hepatitis B infections have become a serious public health issue globally, and the current first-line antiviral treatment for this disease is not a true cure. Recently, sodium taurocholate cotransporting polypeptide (NTCP), a liver-specific bile acid transporter, was identified as a bona fide receptor for hepatitis B virus (HBV) and its satellite virus, hepatitis delta virus (HDV). Identification of the HBV receptor has led to the development of robust cell cultures and provides a potential target for new treatments. This review summarizes the process by which NTCP was discovered and describes its clinical significance as the receptor for HBV and HDV entry.

## Introduction

Chronic hepatitis B (CHB) infection is a major public health problem that affects ~ 250 million people worldwide and can progress to cirrhosis, hepatocellular carcinoma (HCC), hepatic failure, and even death^1^. Thus, there is currently a pressing need to identify a cure for this disease. First-line antiviral treatments currently contain pegylated interferon (PEG-IFN), which is primarily an immunomodulator, as well as nucleos(t)ide analogs that have a high barrier to resistance, such as entecavir, tenofovir disoproxil or tenofovir alafenamide^2,3^. However, these agents do not truly eradicate hepatitis B virus (HBV) because covalently closed circular DNA (cccDNA), which is associated with viral persistence, still remains in hepatocytes, and the host immune response is inadequate^4^.

HBV is a small enveloped virus belonging to the Hepadnaviridae family with a 3.2 kb partially double-stranded genome. The HBV envelope includes three different multi-pass transmembrane proteins, the small (S), medium (M), and large (L) envelope proteins, which share a common C-terminal S domain but have distinct N-terminal domains. The N-terminal extensions of the M and L proteins are referred to as preS2 and preS1/S2, respectively^5^. In contrast, hepatitis D virus (HDV), a satellite of HBV, makes use of HBV surface proteins for its packaging and cellular entry^6^. The process of HBV infection involves viral entry, the formation of cccDNA, reverse transcription, antigen synthesis, and eventually viral release^3^. Furthermore, the virus is believed to bind to a liver cell surface receptor molecule to promote its infection. Given that viral entry is the first step in establishing an infection, numerous attempts have been made to identify the bona fide HBV/HDV receptor to inhibit viral entry into hepatocytes for prevention and treatment.

### Discovery of a bona fide HBV receptor

Over the past few decades, numerous studies have been published related to the identification of the regions of hepatocytes that HBV targets. Neurath et al.^7^ reported that the corresponding ligands of HBV responsible for binding to liver cells are residues 21–47 in the preS1 region of the L protein, corresponding to amino acids 10–36 in genotype D. Subsequently, many studies showed that amino acids 2–48 of preS1 mediated the attachment of the HBV to its target cells, with this region containing essential residues at aa 9–18 in genotype D^6,8–10^. Accordingly, the preS1 peptide 21–47, as described by Neurath et al., is not inhibitory at all, since it lacks an essential asparagine (Asn, N) at position 9 of preS1 (numbering according to genotype D). In contrast, others regarded the 75 N-terminal residues in the preS1 domain as ligands^11,12^. Thus, the range of the potential HBV receptor-binding sites remained unclear. Moreover, the myristoylation of the large surface protein is thought to be essential for viral infection^6,8,9^. On the other hand, the antigenic loop (AGL) of the S protein, apart from the preS1 domain of the L protein, is also related to HBV infection^12,13^. Thus, the AGL of the S protein, and especially myristoylation of the N-terminal preS1 domain in the L protein, are crucial for HBV infection.

A number of molecular structures have been reported as HBV receptor candidates, such as interleukin-6 (IL-6), asialoglycoprotein receptor (ASGPR), and P80^14–16^. Unfortunately, none of these proved to be functional in viral infection. The primary difficulty in the search for HBV-specific receptors is the lack of a convenient in vitro infection system. For a long time, primary cultures of human hepatocytes (PHHs) were the only in vitro model susceptible to HBV and HDV^17^. However, PHHs are difficult to obtain and have a high donor-to-donor variability. Through countless efforts, the HepaRG cell line and primary tupaia hepatocytes (PTHs) were also demonstrated as being susceptible to HBV and HDV infection^18,19^. Subsequently, using PTHs and HepaRG, heparan sulfate proteoglycans (HSPGs) were confirmed as low-affinity HBV receptors^20,21^, which affect the initial binding step of the virus to the target cells mediated by the AGL of the S protein^22^. Notwithstanding this finding, a more specific and high-affinity HBV receptor was sought.

Five years ago, at Peking University, Yan et al.^23^ used a synthesized lipopeptide consisting of the first 2–48 amino acids of the preS1 domain as a probe to identify sodium taurocholate cotransporting polypeptide (NTCP), a bile acid transporter that is primarily expressed on the surface of hepatocytes, as a functional receptor for HBV and HDV using tandem affinity purification and mass spectrometry. At the same time, they showed that residues 157–165 of NTCP were crucial for HBV and HDV binding and infection. Subsequently, other studies confirmed these findings and revealed that residues 84–87 in mouse NTCP (mNTCP) were also vital for HBV entry and infection^24–26^. Recently, residue 263 of NTCP was identified as a novel site that is crucial for viral infection^27^. The results of all of these studies demonstrate that NTCP is the major high-affinity receptor for HBV and HDV.

**Fig. 1:**
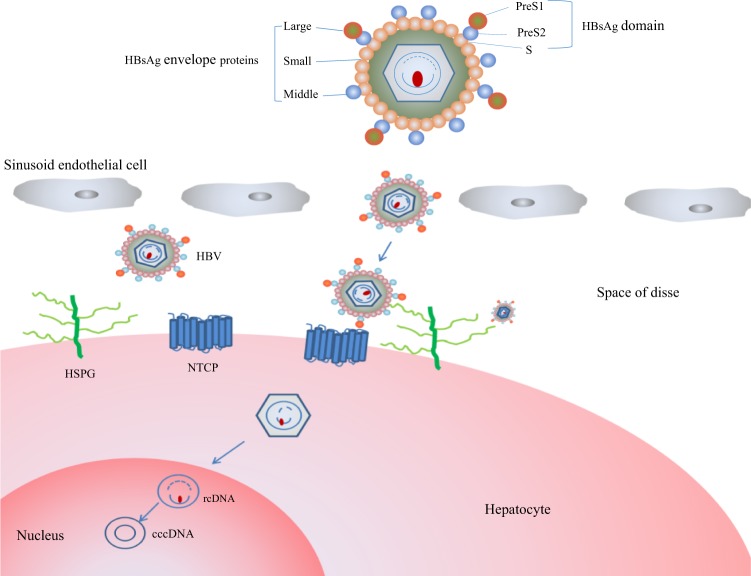
Diagram of the HBV receptor and viral entry. HBV from the blood initially crosses into the liver and attaches to HSPGs, which is followed by the virus binding to the bona fide receptor, NTCP. Next, the HBV virions are uncoated, and the rcDNA is transported into the nucleus, where the highly stable cccDNA serves as a template for viral transcription. HSPG, heparan sulfate proteoglycan; NTCP, sodium taurocholate cotransporting polypeptide; cccDNA, covalently closed circular DNA; rcDNA, relaxed circular DNA

Human NTCP (hNTCP) consists of 349 amino acids and is encoded by the *SLC10A1* gene, which maps to chromosome 14q24.1–24.2^28,29^. NTCP is a multi-transmembrane glycoprotein^30^ that is predominantly expressed on hepatic basolateral membranes and likely crosses the membrane nine times (Fig. 1). Early studies suggested that by cotransporting bile acids with sodium ions at a stoichiometry of 1:2^31^, NTCP is responsible for > 80% of conjugated taurocholate uptake but < 50% of unconjugated cholate uptake from the blood into the liver cells, playing a significant role in the enterohepatic circulation of bile acids^29^.

### Single-nucleotide polymorphisms (SNPs) of NTCP

Recently, substantial genetic research on the relationship between the SNPs of NTCP and HBV infection in humans validated the importance of NTCP in HBV infection at a population level.

The distribution of SNPs in NTCP is dependent on the ethnicity of an individual. The T668C and G190A SNPs, which are variants that are only present in African Americans and Koreans, respectively, have allele frequencies of 5.5% and 1%, respectively, and lead to a decrease in plasma membrane expression of NTCP and taurocholate uptake^32,33^. Previous studies have reported that S267F is a missense mutation that is primarily present in Asian individuals and is present in ~ 3.1–5, 7.4, 7.5, 9.2, and 7.4% of Korean, Chinese, Chinese American, Vietnamese, and Thai individuals^32–35^.

An in vitro experiment showed that the uptake of a bile salt substrate by NTCP is blocked by the myristoylated HBV preS1 domain and vice versa. In addition, mutations in the residues of NTCP that are vital for bile acid binding (N262, Q293, and L294) and sodium binding (Q68A and E257A) were reported to impair bile salt uptake activity and inhibit viral infection^36^, suggesting that HBV/HDV entry and bile salt transport share common NTCP-binding sites and may compete with each other. Moreover, the SNP S267F in NTCP leads to a defect in the transport of bile acids and the loss of the ability to support HBV and HDV infection in cell culture. A larger cohort study of 1899 CHB patients recruited from the Guangdong Province in China showed that the S267F (c.800 C > T, rs2296651) NTCP variant is associated with resistance to chronic hepatitis B and a low incidence of acute-on-chronic liver failure^37^. Conversely, a smaller genetic association study, including 244 CHB patients of the Chinese Han nationality, suggested that this mutation is related to the susceptibility to and chronicity of HBV infection^38^. Another large cohort of 3801 Taiwanese CHB patients also reported that the S267F variant is correlated with resistance to HBV infection and a decreased risk of cirrhosis, and HCC in patients with CHB^39^. Interestingly, heterozygous and homozygous CHB patients were observed in both the Guangdong cohort and the Taiwan study who carried the S267F variant, which is required to lose the ability to promote HBV infection in vitro^36^. The previous study revealed that the heterozygous patients were still susceptible to HBV infection when HepG2 cells were co-transfected with the S267F variant and the wild-type NTCP at a 1:1 ratio^36^. Furthermore, the homozygous patients were infected with HBV, indicating that there may exist more than one pathway for HBV entry or that an adaptation of the virus to the mutated receptor occurred.

A multicenter study in Thailand first demonstrated that the S267F (GA genotype) variant is independently correlated with a continued normalization of alanine transaminase (ALT) after treatment with PEG-IFN within 24 weeks in CHB-infected patients who were HBeAg-positive. Moreover, the patients carrying the S267F variant tended to exhibit a more responsive treatment, effective virological response and HBsAg seroclearance, although the findings did not reach statistical significance^35^. Given that the S267F mutation in NTCP may enhance the antiviral effects of PEG-IFN, it provides a novel assessment for multiple treatment options.

Interestingly, a hospital-based case–control study, which involved 1023 HBV-persistent carriers, 735 people with natural HBV clearance and 732 HBV marker-negative subjects, genotyped three regulatory SNPs (rs8011311, rs7154439, and rs111409076) in *SLC10A1* in a Han Chinese population from central China and uncovered that the common variants were not related to HBV susceptibility in a Chinese population^40^. In contrast, another population-based study, which involved 3650 subjects from eastern China, showed that a functional genetic variant (rs4646287) located in the first intron of NTCP may increase the risk of HBV infection in Han Chinese individuals^41^. These opposing findings may be attributed to different regional distributions of the SNPs. Thus, future studies of this topic will require a larger sample from different geographical areas.

As described above, NTCP is not only crucial for bile salt uptake but also for HBV entry. Therefore, although a given NTCP variant may impact HBV/HDV infection, whether it negatively affects the health of the individuals carrying the mutation by influencing the transport of bile salts is still a cause for concern. An in vivo investigation using NTCP-knockout mice (*Slc10a1*^*−/−*^) showed that these animals had elevated serum bile acids, especially conjugated ones, but no signs of cholestasis, inflammation, or hepatocellular damage were observed^42^. In 2015, a study described a 5-year-old girl from Afghan who was the first patient with a single point mutation (R252H, c.755 G > A) in the *SLC10A1* gene leading to NTCP deficiency, and this individual suffered from a conjugated hypercholanemia (TBA of up to 1500 µmol/L, ref. < 16.3 µmol/L) and exhibited mild hypotonia, growth retardation, and delayed motor milestones. Meanwhile, no clinical signs of cholestatic jaundice, pruritis, or liver dysfunction were observed^43^. After 3 years of follow-up, the girl was still developmentally delayed but not progressive. In addition, although her total bile salt levels remained at a high, there appeared to be a tendency toward lower values. The observation of this phenomenon suggests that NTCP deficiency is a new inborn error of metabolism with an attenuated phenotype^44^. Soon thereafter, another study reported the homozygous S267F (c.800 C > T) variation in a boy at 30.5 months and in a 30-year-old female. The child experienced remarkable hypercholanemia (TBA of up to 737.8 µmol/L) with only mild vitamin D deficiency, mite dermatitis, and transient cholestatic jaundice in the early infant stage. For the adult female, there was only a slight increase in bile acid (19.3 µmol/L)^45^. These findings suggest that NTCP deficiency causes prominent hypercholanemia and clinical symptoms in pediatric patients, particularly in early infancy, but causes a mild increase in TBA for adults, which was also shown in the other report^46^. Moreover, the homozygous S267F mutation in *SLC10A1* is associated with low vitamin D levels as well as differences in steroid hormone and blood lipid levels^47^.

Although NTCP is a primary translocator for conjugated bile acid uptake, there are alternative transport mechanisms through the basolateral membrane of hepatocytes, such as organic anion-transporting polypeptides, the heterodimer OST α/OST β^48,49^, and mEH^50^, which can compensate for the loss of NTCP function. Owing to the mechanism mentioned above, TBA may not increase without limit and could decrease with age in kids that lack a normal NTCP.

#### Regulation of NTCP expression

The expression of NTCP is regulated by different factors, such as bile acid, cytokines, and hormones^51^. NTCP transports the bile salt that is reabsorbed through the portal vein into hepatocytes. However, the increased bile salt levels in the liver cells also inhibits NTCP expression by a negative feedback loop. This phenomenon is due to diverse cellular signaling pathways at the NTCP transcriptional level. At first, cholestasis activates the farnesoid X receptor and then induces the small heterodimer partner (SHP). Once the activity of hepatic nuclear factors 1α and 4α, as well as the expression of the retinoid X receptor, are suppressed by SHP, NTCP is strongly downregulated^42,52,53^. Moreover, several studies have determined that cytokines, especially IL-6, can inhibit HBV entry by downregulating NTCP^54,55^. In addition, hormones have a role in NTCP expression, as a previous study revealed that estrogen inhibits the expression of NTCP in rats. Nevertheless, there is no gender difference in NTCP expression humans^56^. Interestingly, there is a glucocorticoid response element in the promoter region of *SLC10A1*, which may account for the occurrence of HBV infection activation in patients on glucocorticoid therapy^53^. In addition, the retinoic acid receptor (RAR) has been shown to regulate the promoter activity of the hNTCP gene to promote HBV infection^57^. Another experiment has demonstrated that during cell cycle progression, NTCP expression is transcriptionally suppressed by cyclin D1, which is vital for driving cells from the G0/G1 phase to the S phase. Moreover, the decreased expression of NTCP is correlated with a poor prognosis and lower HBV cccDNA levels in HCC patients^58^. These observations show that NTCP may function as a novel prognostic indicator of the survival rate after surgery in HCC patients. Interestingly, not only was NTCP confirmed as a high-affinity receptor for HBV and HDV, but it was also revealed as a host factor that regulates hepatitis C virus infection by augmenting the bile acid-mediated repression of interferon-stimulated genes^59^.

### Identification of NTCP improves HBV cell culture and animal models

In past years, the absence of robust cell culture models and appropriate animal models for HBV infection impeded the in-depth investigation of the association between viral and host factors. For a long time, PHHs were the only available cell culture models to study HBV infection^17^. Although PHHs are the most physiologically relevant in vitro model, they have a short survival time in vitro and are available in limited supply^60^. As PHHs are often obtained from donor liver biopsies, there is a high donor-to-donor gene variability, resulting in a limited a number of reproducible studies^61,62^. Subsequently, PTHs, available from tree shrews, were observed to be more reproducible than PHHs with respect to HBV infection^18^. In 2002, the HepaRG cell line was obtained from a female suffering from liver cancer related to hepatitis C virus^19^. This cell line is a hepatic progenitor cell line possessing a bidirectional differentiation potential, differentiating into hepatocyte-like and cholangiocyte-like cells that exhibit most hepatic functions after induction with dimethyl sulfoxide (DMSO) and hydrocortisone^63^. However, the strict culture conditions and long-term differentiation process of 4 weeks put this cell line at a disadvantage. In addition, all three cell lines mentioned above have a common defect of having low infection efficacy for HBV. Huh7 and HepG2 cell lines lack NTCP expression, are derived from hepatocarcinoma cell lines, and are not susceptible to HBV and HDV infection^64,65^. Interestingly, the transfection of these cell lines with a plasmid encoding HBV DNA or HDV RNA with HBV envelope proteins makes it possible for them to support HBV/HDV infection^11,66^. Nonetheless, the transfection process, but not the natural infection course, cannot provide a comprehensive understanding of the complete viral cycle, in particular, the early stages of viral absorption, entry, and uncoating, etc. Notably, in the context of the identification of NTCP as the specific receptor for HBV and HDV infection, the exogenous expression of hNTCP in Huh7 and HepG2 cell lines confers susceptibility to HBV and HDV infection^23,24,67^, providing a more robust and efficient in vitro model to study the full viral cycle and virus-host interactions. Moreover, NTCP-overexpressing hepatoma cell lines are productive tools to screen for antiviral molecules targeting cell entry. However, these cell lines still have limitations in that they partially mimic physiological hepatic functions, a high multiplicity of infection (MOI) and polyethylene glycol (PEG) are needed, and they synthesize low amounts of cccDNA^68^. Taken together, the currently available cell cultures share common disadvantages. For instance, they require the use of molecular adjuvants (DMSO or PEG) for infection, have high MOIs and have a low replication efficiency. Recently,  in-vitro hepatogenic differentiation umbilical cord matrix stem cells (D-UCMSCs), which are susceptible to HBV, were used as a non-transformed in vitro model to study the early stages of viral transmission due to their ability to endogenously regulate the expression of hNTCP. Besides, although D-UCMSCs also need high MOIs to enhance efficient infection, they are closer to the physiological conditions and do not require molecular adjuvants^69^. Furthermore, the expression of NTCP in D-UCMSCs serves both as an indicator of viral entry and as a novel marker of stem cell hepatogenic differentiation quality.

HBV shows remarkable species specificity, only infecting humans, chimpanzees, and tree shrews (*Tupaia belangeri*)^70^, whereas other animals, such as mice, rats, and macaques, etc., are resistant to HBV and HDV infection^71^. Many in vitro and in vivo experiments have shown that in macaques, NTCP does not bind the preS1 lipopeptide, owing to the region at amino acids 157–165 of hNTCP, which has been shown to be crucial for binding preS1 and HBV infection^23,24,72^. Curiously, a naturally occurring transmissible chronic HBV infection (genotype D) was reported among *Macaca fascicularis* from Mauritius Island, although the viral load was exceedingly low^73^. Whether HBV has a complete viral cycle or marginal replication levels in these animals remains an open question. At present, woodchuck hepatitis virus, one of five species of Orthohepadnavirus belonging to the Hepadnaviridae family, supports HBV and HDV infection in HepG2 cells and is mediated by woodchuck NTCP, albeit to a lower extent than hNTCP (~ 90% reduction). Simultaneously, using site-directed mutagenesis, residue 263 of NTCP has been identified as a novel site that is crucial for viral infection and can be exploited as a potential target for blocking viral entry^27^.

In past decades, the chimpanzee was the only immunocompetent animal model for HBV infection. However, because of ethical issues, chimpanzees are not allowed as experimental animals anymore^74^. Accordingly, there is an immediate need for surrogate animal models for experimentation. Previous investigations revealed that the expression of hNTCP in mouse, rat, and dog hepatocytes encourages the establishment of HDV infection but does not support HBV infection^24,25,72^. Nevertheless, when amino-acid residues 84–87 in mNTCP were substituted by human counterparts, viral infection was effectively supported^25^. These studies suggested that additional host factors, apart from NTCP, may be involved in HBV infection. A more recent report observed that hepatocytes from cynomolgus macaques, rhesus macaques, and pigs become completely susceptible to both HBV and HDV infection after hNTCP transduction^74^. This finding highlights that the endogenous NTCP homologs of these species are the only factor efficiently mediating viral entry, in contrast to murine or rat models. In light of this finding, these novel animal models can be further developed to study HBV infection in vivo.

### Development of HBV entry inhibitors with the discovery of NTCP as a bona fide HBV/HDV receptor

With the recent discovery of NTCP as a bona fide receptor for HBV/HDV, molecular compounds have been identified that inhibit the cellular entry of HBV and/or HDV, which summarized in Table 1.

**Table 1:** HBV/HDV entry inhibitors reported so far

Molecular compound	Category	Antiviral activity (IC50)			Cell culture		NTCP transporter activity (IC50)	Reference
		HBsAg	HBeAg	HDV	HBsAg/HBeAg	HDV		
Myrcludex B	Myristoylated lipopeptide	669 pM	83 pM	NA	PHH	NA	52.5 nM	^78^
		140 pM	NA	NA	HepaRG	NA	NA	^10^
CsA	Immunosuppressant	1.17±0.22 µM	<4 µM	NA	HepaRG	NA	1.12 µM	^77^
		372 nM	311 nM	4–8 µM	PHH	HuH7-hNTCP	3.3 µM	^78^
		1.25 µM	NA	NA	HepG2-hNTCP-C4	NA	NA	^81^
		<8 µM	1.27±0.43 µM	NA	HepG2-hNTCP-C4/PHH	NA	1.49±0.1 µM	^79^
CsA derivatives	Nonimmunosuppressant							
SCYX618806		0.43±0.12 µM	NA	NA	HepaRG	NA	NA	^77^
SCYX827830		0.38±0.08 µM	NA	NA	HepaRG	NA	NA	^77^
SCYX1454139		0.17±0.02 µM	NA	NA	HepaRG	NA	NA	^77^
SCY806		<8 µM	1.05±0.33 µM	NA	HepG2-hNTCP-C4/PHH	NA	>10 µM	^79^
SCY446		<8 µM	0.54±0.13 µM	NA	HepG2-hNTCP-C4/PHH	NA	1.92±0.08 µM	^79^
SCY450		<8 µM	0.91±0.19 µM	NA	HepG2-hNTCP-C4/PHH	NA	>10 µM	^79^
SCY995		<8 µM	1.97±0.04 µM	NA	HepG2-hNTCP-C4/PHH	NA	>25 µM	^79^
CsB	Immunosuppressant	0.8 µM	NA	NA	HepG2-hNTCP-C4	NA	NA	^81^
UDCA	Non-conjugated bile acid	<100 µM	<100 µM	NA	HepaRG	NA	NA	^77^
		<100 µM	NA	NA	HepG2-hNTCP-C4	NA	NA	^81^
		<25 µM	20 µM	NA	HepaRG/HepG2-NTCP	NA	NA	^36^
TUDCA	Conjugated bile acid	NA	NA	NA	NA	NA	<10 µM	^77^
		<25 µM	0.37–1.11 µM	NA	HepaRG/HepG2-NTCP	NA	1.1–3.3 µM	^36^
Progesterone	Sex hormone	<40 µM	<40 µM	NA	HepaRG	NA	NA	^77^
		<40 µM	NA	NA	HepG2-hNTCP-C4	NA	NA	^81^
Bromosulfophthalein	For liver function test	<100 µM	NA	NA	HepG2-hNTCP-C4	NA	NA	^81, 95^
22(S)-hydroxycholesterol	Oxysterol	<11 µM	NA	NA	HepG2-hNTCP-C4	NA	NA	^81^
		0.3–0.9 µM	NA	NA	HepaRG	NA	NA	^81^
Ezetimibe	Cholesterol absorption inhibitor	18 µM	18 µM	NA	HepaRG	NA	NA	^83^
		NA	NA	<10.4 µM	NA	Huh7-hNTCP	NA	^88^
Irbesartan	Angiotensin II receptor inhibitor	35v	35 µM	NA	HepG2-NTCP	NA	NA	^87^
		NA	NA	<9.4 µM	NA	Huh7-hNTCP	NA	^88^
		17–50 µM	6–17 µM	50–150 µM	HepaRG	HepaRG	NA	^86^
Ritonavir	HIV protease inhibitor	NA	NA	<9.1 µM	NA	Huh7-hNTCP	NA	^88^
EGCG	Green tea polyphenol	10–20 µM	NA	NA	HuS-E/2	NA	NA	^85^
Vanitaracin A	Fungal tricyclic polyketide	0.61±0.23 µM	<45 µM	<6.25 µM	PHH/HepG2-hNTCP-C4	HepG2-hNTCP-C4	2–4 µM	^84^
Ro41-5253	Antagonist of retinoic acid receptor	5–10 µM	<100 µM	NA	HepaRG	NA	NA	^57^
Interleukin-6	Cell factor	<0.15 ng/ml	NA	NA	HepaRG	NA	0.15–0.62 ng/ml	^55^
		0.15–0.62 ng/ml	NA	NA	PHH	NA	2.5–10 ng/ml	^55^
PAC	Oligomeric flavonoid	7.8±0.75 µM	NA	<20 µM	PHH	PHH	>20 µM	^91^
OHBF-C	Oligomeric flavonoid	4.3±1.2 µM	NA	NA	HepG2-hNTCP-C4	NA	NA	^91^
Rosiglitazone	PPARγ agonists	17–50 µM	6–17 µM	17–50 µM	HepaRG	HepaRG	5.1 µM	^86, 96^
Zafirlukast	Leukotriene receptor antagonist	17 µM	6–17 µM	17–50 µM	HepaRG	HepaRG	6.5 µM	^86, 97^
TRIAC	Suppress pituitary secretion of TSH	17–50 µM	6–17 µM	17–50 µM	HepaRG	HepaRG	6.9 µM	^86, 98^
Sulfasalazine	Immunosuppressant	17–50 µM	17–50 µM	50–150 µM	HepaRG	HepaRG	9.6 µM	^86, 99^
Chicago sky blue 6B	VGLUTs inhibitor	6–17 µM	6–17 µM	17 µM	HepaRG	HepaRG	7.1 µM	^86, 100^
2H5-A14	Humanized monoclonal antibody	<50 pM	<50 pM	NA	HepG2-hNTCP-C4	NA	NA	^92^
Bosentan	Endothelin-receptor antagonist	<100 µM	<100 µM	NA	HepaRG	NA	NA	^77^
Propranolol	β-receptor blocker	100 µM	NA	NA	HepaRG	NA	NA	^77^

*CsA* cyclosporin A, *CsB* cyclosporin B, *UDCA* ursodeoxycholic acid, *TUDCA* tauroursodeoxycholic acid, *EGCG* epigallocatechin-3-gallate, *PAC* proanthocyanidin, *OHBF-C* oolonghomobisflavan C, *HIV* human immunodeficiency virus, *PPARγ* peroxisome proliferator-activated receptor gamma, *TSH* thyroid ttimulating hormone, *VGLUT* vesicular glutamate transporter, *HBV* hepatitis B virus, *HDV* hepatitis delta virus, *PHH* primary cultures of human hepatocyte, *NTCP* sodium taurocholate cotransporting polypeptide, *IC50* half maximal inhibitory concentration, *NA* not applicable

Although HSPG was shown to be a low-affinity receptor for HBV in the initial binding process of the virus to the host cell, a detailed mechanism remains to be elucidated. Glypican 5 (GPC5), a subclass of HSPGs, was recently demonstrated as an attachment factor and an antiviral target for HBV and HDV entry by NTCP-based cell culture systems^66^. Thus, a monoclonal antibody targeting GPC5 can be utilized to inhibit HBV infection.

As the NTCP inhibitor with the most potential, Myrcludex B, a myristoylated preS1-derived peptide^8^, has been evaluated in clinical trials. A phase I clinical trial, which included 36 healthy people, revealed that Myrcludex B was well tolerated, even when applied at doses of up to 20 mg intravenously. In contrast, the bioavailability of Myrcludex B when injected subcutaneously was greater (85%). A pharmacokinetic model showed that a subcutaneous dose above 10 mg led to a target saturation of > 80% for at least 15 h^75^. In a phase 2a clinical trial, the safety and tolerability of Myrcludex B were assessed. In one cohort, 40 chronic hepatitis B patients, who were HBeAg-negative (HBV DNA > 2000 IU/ml, median HBV DNA 4.7 log10 IU/ml, without cirrhosis), were treated for 12 weeks with daily doses of Myrcludex B at that ranged from 0.5 to 10 mg. In addition, the patients who received 10 mg were had their treatment extended to 24 weeks. Of note, in the 10 mg group, the HBV DNA was reduced by > 1 log10 at week 12 in six of the eight patients (75%), whereas a lower response rate (7/40; 17%) was observed in the rest of dosed groups. Moreover, normal levels of ALT were achieved in 22/40 (55%) patients, but no significant impact on patient HBsAg levels was observed. In another cohort, 24 patients infected with HDV were divided into three eight-person groups and were treated for 24 weeks with daily doses of 2 mg of Myrcludex B, alone or in combination with PEG-IFNα or with PEG-IFNα monotherapy. Consequently, HDV RNA levels decreased by > 1 log10 in all the subgroups at week 24. Remarkably, HDV RNA became negative in 5/7 patients administered the combination therapy, whereas this was observed 2/7 patients in the Myrcludex B monotherapy group at week 24, indicating that the drug combination was a more effective at treating HDV. Moreover, the patients taking Myrcludex B exhibited no serious adverse effects, with only a slight increase in bile acid levels (Bogolomov et al., AASLD meeting 2014). Thus, Myrcludex B was safe and well tolerated in chronic hepatitis B patients with or without HDV coinfection. In addition, another recent experiment involving 12 healthy volunteers assessed the coadministration of Myrcludex B with tenofovir, showing that Myrcludex B induced a remarkable increase in bile acid concentrations without causing any relevant symptoms and had no influence on tenofovir pharmacokinetics^76^. Currently, tenofovir, a nucleos(t)ide reverse transcriptase inhibitor, is a first-line antiviral. The study indicated that Myrcludex B may provide an add-on treatment to tenofovir, but further research is still needed. Remarkably, Professor Heiner Wedemeyer, from Hannover Medical School in Germany, announced the final results of a Phase 2b clinical trial (MYR 202), investigating a combination of Myrcludex B and tenofovir disoproxil fumarate (TDF) in chronic hepatitis B patients co-infected with HDV. The study recruited 120 volunteers in 20 centers from Russia and Germany. The 120 subjects were randomly divided into four treatment groups. Three groups received a pretreatment of 245 mg of TDF for at least 12 weeks, after which Myrcludex B was administered at doses of 2 mg (A), 5 mg (B), or 10 mg (C) subcutaneously once daily for 24 weeks, followed by TDF alone until week 48. During the entire 48 weeks, group D was administered TDF alone. The primary endpoint was that HDV RNA decreased > 2 log10 or became negative. After 24 weeks of Myrcludex B plus TDF, 46.4, and 46.8% of individuals on the 2 and 5 mg doses reached the endpoint, respectively, increasing to 76.6% for those treated with the 10 mg dose. In contrast, the serum HDV RNA levels showed no significant decrease in the TDF monotherapy group (only 3.3%). The median decrease in HDV RNA were 1.75 log10 (A), 1.6 log10 (B), and 2.7 log10 (C) versus 0.18 log10 in the control group. Meanwhile, a significant trend in ALT normalization was observed in the Myrcludex groups. Nevertheless, the HDV RNA levels and ALT normalization soon rebounded after Myrcludex B was discontinued. Furthermore, the optimal duration of treatment and the possible combination therapies need to be determined. Wedemeyer suggested that 2–3 years of treatment with Myrcludex may be needed to eliminate HDV RNA. During the treatment process, Myrcludex B demonstrated a favorable safety profile, with no drug-related severe adverse events or treatment discontinuations owing to the agent. Although 50, 74% and 91% of individuals taking the 2, 5, and 10 mg doses had increased bile acids, respectively, compared with 19% in group D, none of the participants reported pruritus or itching. Moreover, the trend of a notable decrease in liver stiffness was observed in the Myrcludex groups (Wedemeyer et al., EASL meeting April 2018).

Cyclosporin A (CsA) is a cyclic peptide of 11 amino acids that serves as an immunosuppressant to treat autoimmune diseases and is administered after organ transplantations. CsA was known to be an NTCP inhibitor before NTCP was described as an HBV receptor. By binding to cyclophilins and, consequently, inhibiting calcineurin, CsA has immunosuppressive activity that may promote viral infection or replication in patients. Therefore, it is noteworthy that immunosuppression was eliminated from new agents. Several studies have reported that CsA and its derivatives, independent of binding to cyclophilin and calcineurin, can interfere with HBV entry by blocking the interaction between HBV preS1 and NTCP^77–79^. Notably, CsA analogs, particularly SCYX1454139, were identified with higher anti-HBV potency than CsA^77^. Similar to Myrcludex B, the half maximal inhibitory concentration (IC50) of CsA for inhibiting HBV/HDV infection is far below that of its taurocholate uptake inhibition levels^77,78^. In other words, there is a therapeutic concentration range that largely inversely inhibits HBV infection, which has little impact on the transport of bile salts. Subsequently, four other CsA derivatives, SCY806, SCY446, SCY450, and SCY995, also showed high anti-HBV activity with no immunosuppressive activity. Intriguingly, SCY806, SCY450, and SCY995 had no impact on the NTCP-dependent uptake of bile acid, whereas SCY446 caused a significant reduction in bile acid uptake^79^. Nevertheless, CsA inhibits NTCP as well as other transporters, such as the organic anion transporting polypeptide, at even lower concentrations^80^, which may lead to a higher risk of cholestasis compared Myrcludex B. As a consequence, the usefulness of CsA as an agent to treat HBV and HDV infection remains to be seen.

Endogenous substrates of NTCP, such as ursodeoxycholic acid, tauroursodeoxycholic acid, progesterone, bromosulfophthalein, and 22(S)-hydroxycholesterol^26,36,77,81^, can also block HBV/HDV entry by interacting with the bile acid pocket of NTCP. Ezetimibe is a selective inhibitor of intestinal cholesterol absorption to block lipid transport and is also a substrate of NTCP^29,82^ that has been shown to interfere with HBV and HDV entry^83^. Intriguingly, vanitaracin A, a novel tricyclic polyketide identified from a fungal secondary metabolite library, also targets NTCP to inhibit HBV/HDV infection^84^.

Other strategies do not directly block NTCP but inhibit viral entry by altering the expression or interaction of the endocytosis/fusion of NTCP. A small screen of several common phytochemicals identified epigallocatechin-3-gallate (EGCG) as a novel inhibitor of HBV entry. EGCG potently inhibits HBV entry via inducing clathrin-dependent endocytosis/fusion of NTCP from the cell membrane followed by protein degradation, which can be applied to prevent the reinfection of HBV^85^. In another study, Ro41-5253, an antagonist of the RAR, which regulates the promoter activity of the hNTCP gene, inhibits HBV entry by reducing NTCP expression^57^. In addition, IL-6 inhibits HBV entry by downregulating NTCP expression^54^.

Recently, five out of 1280 clinically approved drugs, i.e., rosiglitazone (IC50, 5.1 µM), zafirlukast (IC50, 6.5 µM), TRIAC (IC50, 6.9 µM), sulfasalazine (IC50, 9.6 µM), and Chicago sky blue 6B (IC50, 7.1 µM), were shown to inhibit NTCP-mediated bile acid uptake. At a similar concentration, the five compounds inhibit bile acid transport to block HBV/HDV infection (IC50, 5–10 µM) in a dose-dependent manner in vitro^86^. Indeed, rosiglitazone, zafirlukast, and sulfasalazine are more specific for NTCP, whereas TRIAC and Chicago sky blue 6B are equally effective against NTCP and apical sodium bile acid transporte (ASBT), which is predominantly expressed at the brush border of ilealenterocytes and also mediates cellular uptake of conjugated bile acids. Moreover, Chicago sky blue 6B also inhibits vesicular glutamate transporters, with no connection with bile acid transportation, further demonstrating its low specificity. Other FDA-approved drugs, including cyclosporine B, propranolol, bosentan, ritonavir, and irbesartan, have also been verified as NTCP inhibitors for their potency in preventing HBV/HDV infection^77,81,87–89^.

It must be noted that all the agents mentioned above that target NTCP can affect bile acid uptake into hepatocytes, which may cause side-effects. Although Myrcludex B has a therapeutic concentration range that inhibits HBV infection, it also marginally impairs NTCP transporter activity. In fact, the L protein preS1 domain at the N-terminus is crucial for HBV in addition to its cellular receptor, namely, NTCP. Some studies have examined new strategies for targeting the L protein preS1 region, which may inhibit HBV entry without influencing the transporter activity of NTCP. Proanthocyanidin (PAC), a polymer of flavonoid molecules^90^, targets the 2–48 aa of the preS1 region in the large HBV surface protein, which is the most important region of this protein, and in combination with NTCP has been identified as an entry inhibitor of HBV. Oolonghomobisflavan C, an analog of PAC, possesses a stronger anti-HBV activity than PAC. Intriguingly, both of these compounds impair HBV infectivity without dysregulating NTCP-mediated bile acid transport activity^91^. In addition, 2H5-A14, a human monoclonal antibody, has been recently developed to block HBV and HDV infection by targeting the preS1 region, including the NTCP-binding site^92^. In the past, two anti-preS1 neutralizing antibodies, BX-182, and KR127, were used to prevent HBV infection, although neither were able to treat an established HBV infection^93,94^. However, 2H5-A14 can be used as a novel therapy to both prevent and treat HBV and HDV infections^92^. Notwithstanding, these novel agents require additional investigation and validation.

## Conclusion

Chronic hepatitis B infections are still a cause for concern owing to various uncontrollable complications. A number of attempts have sought to target the different stages of the HBV replication cycle in the search for a true cure. As a result, NTCP, which is also responsible for bile acid uptake, has been identified as the bona fide receptor for HBV and HDV infection, providing a new strategy to target the first step of viral entry. Subsequently, studies have identified molecular compounds that inhibit NTCP to varying degrees, most of which simultaneously affect bile acid uptake. Therefore, a novel treatment that targets NTCP but has only a slight influence on bile acid uptake would be more clinically appropriate. Myrcludex B, one of the most promising NTCP inhibitors, is currently undergoing clinical trials and has a therapeutic concentration range that inhibits HBV infection but marginally impairs NTCP transporter activity. In addition, the discovery of the receptor has led to the development of cell culture models and animal models for HBV infection. Therefore, the new perspectives discussed here regarding NTCP as the high-affinity receptor for HBV will help to provide new treatment approaches and a novel platform for an in-depth investigation into its association with viral and host factors.
